# Paraneoplastic Necrotizing Myopathy Post Lumpectomy and Chemotherapy for Early Breast Cancer

**DOI:** 10.7759/cureus.10916

**Published:** 2020-10-12

**Authors:** Priyanka Venkatesh, Sophia M Hitchcock, Jamie Jacobsohn, Anup Kasi

**Affiliations:** 1 Medical Oncology, University of Kansas Medical Center, Kansas City, USA; 2 Internal Medicine, Kempegowda Institute of Medical Sciences/Rajiv Gandhi University of Health Sciences, Bangalore, IND; 3 Neuropathology, Therapath Neuropathology, New York, USA

**Keywords:** idiopathic inflammatory myopathy, paraneoplastic syndromes, necrotizing autoimmune myopathy, breast cancer, creatine kinase

## Abstract

Our case report describes a 60-year-old female patient with a past medical history of Stage IB breast cancer, status post lumpectomy and adjuvant chemotherapy, admitted to our hospital with the chief complaints of fever, myalgia, and muscle weakness. A physical exam revealed proximal muscle weakness and a facial rash. A full workup was done, and the muscle biopsy showed evidence of a necrotizing myopathic process, which confirmed our diagnosis. This led to a diagnosis of necrotizing myopathy, deemed to be paraneoplastic after other possible differentials were ruled out. The patient showed improvement after a five-day course of intravenous immunoglobulin (IVIG) and high-dose steroids. Necrotizing myopathy, as a paraneoplastic process, has been scarcely described. In the context of our case, we review the characteristics and relevant existing literature about paraneoplastic necrotizing myopathy as well as emphasize the need to include it as a differential in the setting of malignancy.

## Introduction

Immune-mediated necrotizing myopathy (IMNM) is a type of idiopathic inflammatory myopathy [1]. It is a rare entity that typically presents with severe proximal muscle weakness, myofiber necrosis with minimal inflammatory infiltrate on muscle biopsy, and infrequent extra-muscular involvement [2]. IMNM has been found in association with viral infections, connective tissue diseases, malignancy, and certain toxins [3]. As a paraneoplastic process, necrotizing myopathy has been described in the setting of gastrointestinal tumors, small cell lung cancer, and breast cancer, among others [4]. We report a very rare case of paraneoplastic necrotizing myopathy manifesting after the treatment of early-stage breast cancer (lumpectomy and adjuvant chemotherapy).

## Case presentation

A 60-year-old woman with a past medical history significant for hypertension underwent a core biopsy for a palpable breast lump measuring 2 x 2 cm that revealed a Grade 2 invasive ductal carcinoma (IDC). Immunohistochemistry (IHC) staining revealed the following hormonal receptor status: estrogen receptor (ER) 100%/progesterone receptor (PR) 20%/human epidermal growth factor 2 (HER-2) negative with a proliferation index marker (Ki-67) of 47%. Subsequently, a lumpectomy and histopathological study of the surgical specimen confirmed the presence of IDC with clear margins and 1/5 nodes with micrometastases. HER-2 was 2+ (equivocal) on IHC of the lumpectomy specimen, which was later confirmed by fluorescence in situ hybridization (FISH) to be negative. The staging workup led to the final diagnosis of stage IB (pT2pN1mi(sn)M0 ER/PR + HER-2 negative) breast cancer. Given her Oncotype Dx score of 31, she was considered high risk and received four cycles of chemotherapy with cyclophosphamide and docetaxel. She also received post-lumpectomy radiation and was started on hormonal therapy with anastrozole. Two weeks later, the patient presented to another facility with persistent low-grade fever, progressive weakness of upper and lower extremities, myalgia, and dyspnea. She was transferred to our hospital with an elevated creatine kinase level, possibly indicating rhabdomyolysis from an underlying inflammatory myopathy. She reported progressive difficulty climbing or descending stairs and brushing her hair, pointing to proximal muscle weakness, which started about a week after the last cycle of chemotherapy. Physical exam showed the weakness of bilateral lower extremities - hip flexors (3/5), hip adductors (4/5), hip abductors (4+/5), left upper extremity - shoulder abduction (4+/5), and elbow extension (4/5) and left-hand weakness - finger flexion (4/5). The patient also developed a rash over her upper eyelid and a malar rash during the course of her hospital stay.

An extensive workup was performed to determine the cause of the rhabdomyolysis. Creatine kinase (CK) was significantly elevated at 17,265 U/L on admission. A comprehensive metabolic panel revealed elevated liver enzymes, which was worked up further and deemed to be due to possible past infection with hepatitis B, an incidental finding. The patient was found to have high immune titers for hepatitis B. The thyroid profile was within normal limits. The autoimmune workup performed as recommended by rheumatology was negative for the following antibodies: antinuclear; anti-Sjögren's-syndrome type A (anti-SSA); Anti-Sjögren's syndrome type B (anti-SSB); anti-Smith; anti-double-stranded deoxyribonucleic acid (DNA); antiproteinase-3; anti-myeloperoxidase; anti-scl-70; anti-ribonucleic acid (anti-RNA) polymerase-III; and anti-centromere antibodies. The rheumatoid factor was also negative. C3 and C4 complement levels were within the reference range. Mayo MyoMarker panel-3 (Mayo Clinic Laboratories, Rochester, MN), testing for 16 different myositis markers was negative (anti-Jo-1, anti-PL7, anti-PL12, anti-EJ, anti-OJ, anti-signal recognition peptide (anti-SRP), anti-Mi-2, anti-TIF1 Gamma, anti-MDA-5, anti-NXP-2, anti-PM/Scl-100, anti-Fibrillarin/U3 RNP, anti-U2 snRNP, anti-Ku, anti-SS-A).

**Figure 1:**
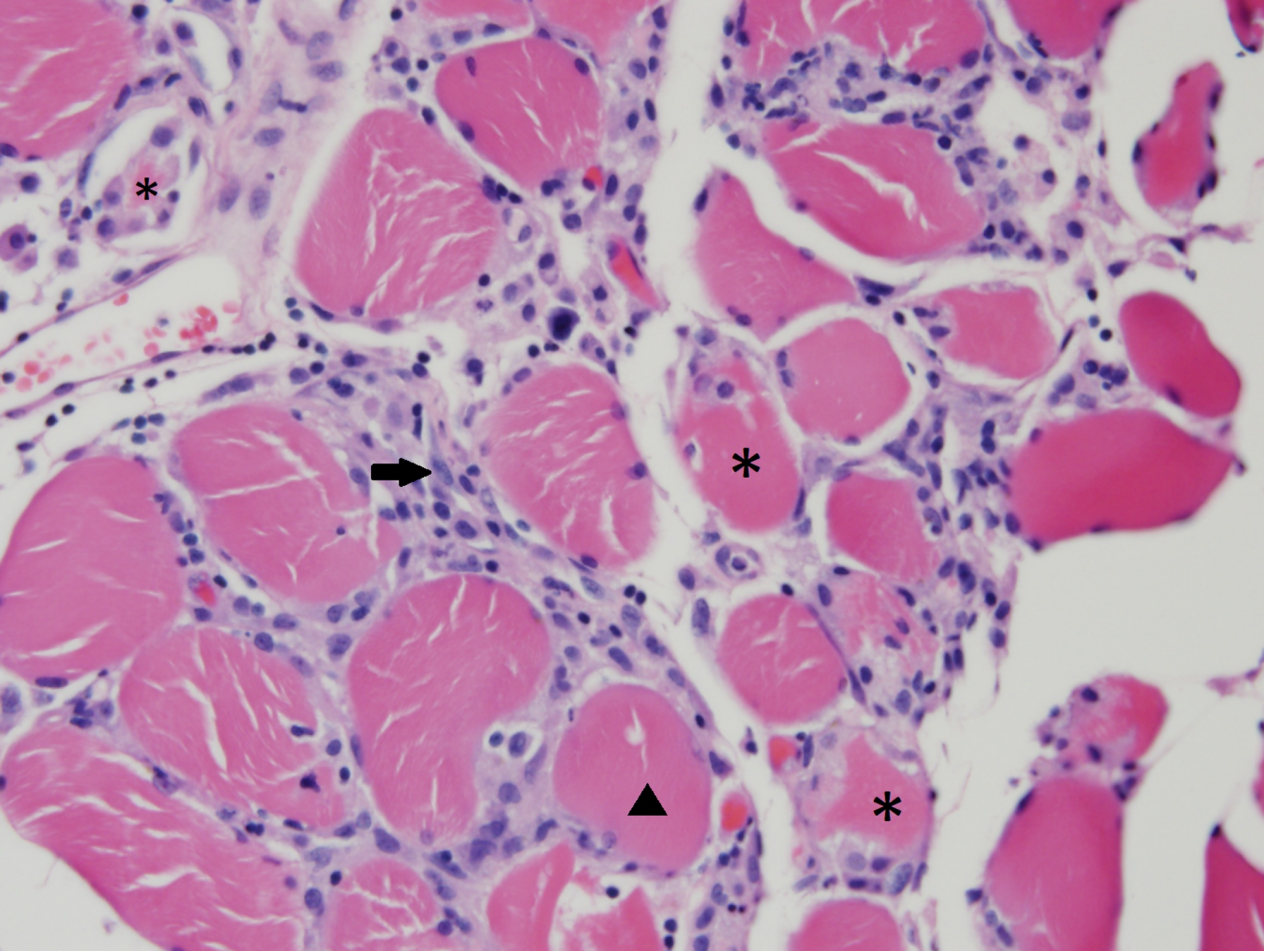
Necrotizing myopathy Photomicrograph of muscle biopsy (right quadriceps femoris). Formalin-fixed. Paraffin-embedded tissue. Hematoxylin and Eosin stain. 400x magnification Skeletal muscle with an increased variation in myofiber size (arrowhead), a few necrotic fibers undergoing myophagocytosis (asterisks), and numerous endomysial macrophages (arrow). There is a marked absence of endomysial or perivascular chronic inflammation.

For the left-hand weakness, neuromuscular specialists recommended magnetic resonance imaging (MRI) of the cervical spine, which was unremarkable, and an MRI of the left brachial plexus to rule out injury from a previous sentinel node biopsy. The MRI showed a normal brachial plexus with edema of the rhomboid major and subscapularis muscles. Electromyography (EMG) revealed evidence of an irritative, proximal myopathy and mild to moderate distal, axonal, sensorimotor polyneuropathy. A muscle biopsy of the right quadriceps muscle was performed and deemed to be an adequate sample. It revealed a necrotizing myopathic process without evidence of significant inflammation. There were marked infiltrates of macrophages in the endomysium without any significant lymphocytic infiltrates. The immunohistochemical stain for major histocompatibility complex (MHC) Class I showed positive staining of the mononuclear cell infiltrates in the endomysium and mild staining of the myofibers. The stain for membrane attack complex (MAC) C5b-9 showed positive staining of the infiltrates in the endomysium and dark segmental staining of scattered myofibers (Figure 1). Oncology workup included a computed tomography (CT) scan of the chest/ abdomen/pelvis, which was negative for metastasis and new primary malignancies.

**Figure 2:**
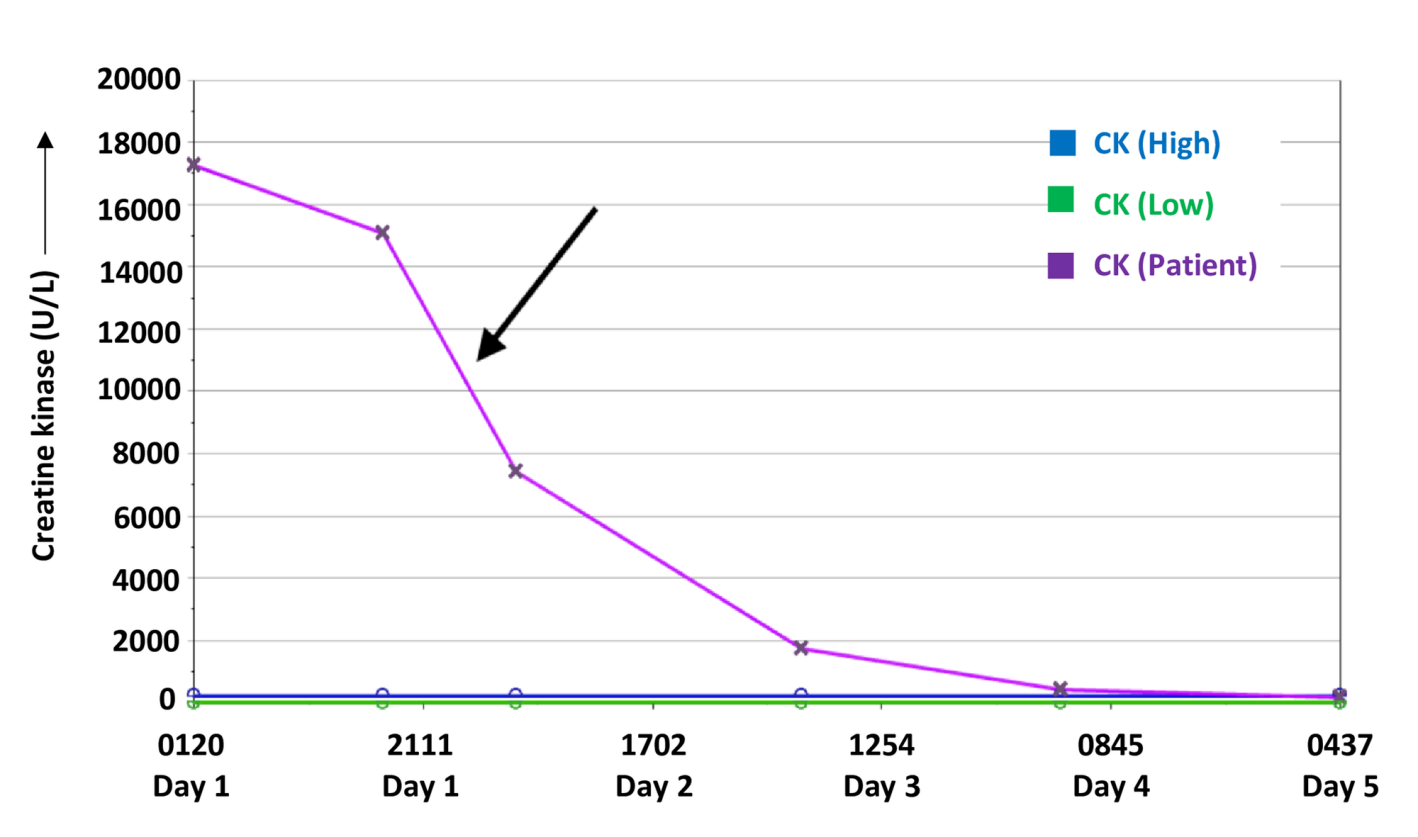
Graph of creatine kinase (CK) levels Graph showing the trend of creatine kinase during the in-patient course in the hospital. There was a marked decline in the CK levels of the patient following the administration of steroids (arrow).

The patient received continuous intravenous (IV) fluids to manage her elevated CK level. Once the diagnosis was made, she was started on intravenous immunoglobulin (IVIG), 0.4 g/kg/day for five days, along with methylprednisolone, 1 g/day for five days. Following the five-day course of IVIG and high-dose steroids, she was transitioned to oral prednisone, 60 mg daily. CK levels continued to decline (Figure 2). The patient was discharged on a month-long course of methotrexate, 15 mg weekly for four weeks. At the time of discharge, the patient reported an improvement in muscle strength and minimal skin manifestations.

The patient has reported feeling well on subsequent visits, with no muscle weakness, fever, or rash since discharge. The patient was scheduled to have outpatient follow-up appointments with rheumatology, neuromuscular, and oncology. Neuromuscular recommended a taper of oral prednisone. Methotrexate was discontinued because of leukopenia. From the oncology perspective, the patient is being followed with tumor marker CA 27-29 and has not shown any recurrence in her follow-up visits. She also did not show any new hypermetabolic lesions on a fluorodeoxyglucose-positron emission tomography (FDG-PET) scan. She has successfully completed post-lumpectomy radiation and continues to take an aromatase inhibitor for hormonal therapy with a goal to continue for five years.

## Discussion

In the setting of proximal muscle weakness with elevated CK enzyme levels, the main differentials considered initially were inflammatory, infectious, or autoimmune myopathy. The workup did not demonstrate any specific infectious etiology. This favored an inflammatory myopathy related to the neoplastic process. The primary considerations were dermatomyositis/polymyositis/inclusion body myositis versus an idiopathic unspecified myopathy. Although the patient had asymmetric muscle weakness, inclusion body myositis (IBM) seemed unlikely, as it was not a chronic progressive weakness involving the distal muscles, as is the usual presentation of IBM. The appearance of a facial rash after admission initially favored dermatomyositis over polymyositis and an unspecified myositis. Myositis-specific antibodies for dermatomyositis, polymyositis, or anti-synthetase syndrome were negative based on the results of MyoMarker Panel-3. As we did not suspect necrotizing myopathy at that time, anti3-hydroxy-3-methylglutaryl-CoA reductase (anti-HMGCR) antibodies were not checked. The MyoMarker panel did, however, include anti-SRP antibodies, which are seen in a subset of patients with IMNM. EMG and MRI findings were consistent with those seen in a myopathic process. The muscle biopsy from the right quadriceps established the diagnosis of necrotizing myopathy. Although a few patients with dermatomyositis do demonstrate necrosis as a feature in the biopsy, the absence of the classical features of dermatomyositis, such as perimysial inflammation, perifascicular atrophy, and perifascicular elevation of MHC class 1, binding of complement to capillaries and the surface of the sarcolemma, and the reduction of capillaries [5] ruled out this diagnosis. Finally, other causes of myopathy, such as endocrine disorders, electrolyte disturbances, drugs, and toxins, were ruled out based on history and laboratory investigations. Chemotherapy-induced myositis was considered given the presentation two weeks after the completion of taxane-based treatment. In all previously reported cases, a temporal relationship with treatment along with elevated CK and muscle weakness was considered sufficient for diagnosis. No biopsy reports were available for these cases [6]. We ruled out chemotherapy-induced myopathy based on the gross elevations in CK, characteristic of IMNM, and the conclusive muscle biopsy report. Other causes of CK elevations were also excluded from the differential. Although the possibility of a de-novo autoimmune process could not be ruled out completely, the appearance of these features in a patient with invasive ductal carcinoma less than a month after completion of treatment makes a paraneoplastic process more likely. A review by Fanous and Dillon describes paraneoplastic neurological manifestations in a patient with early-stage breast cancer, two years after mastectomy [7].

The idiopathic inflammatory myopathies (IIMs) are a heterogeneous group of disorders characterized by muscle weakness, elevated muscle enzymes, autoantibodies, and inflammatory muscle biopsies [2]. The current classification proposed based on the European League Against Rheumatism (EULAR)/American College of Rheumatology (ACR) guidelines published in 2017 include dermatomyositis (DM), juvenile dermatomyositis (JDM), and polymyositis (PM), which includes immune-mediated necrotizing myopathy (IMNM), amyopathic dermatomyositis (ADM), and inclusion body myositis (IBM) [8]. In a large recent analysis of 3067 patients from the Euromyositis registry, it was found that necrotizing myopathy was the second most common entity after dermatomyositis, representing about one-fifth of all the cases. In general, females were found to be more commonly affected [9]. Immune-mediated necrotizing myopathy typically presents with acute or subacute proximal weakness of the arms and legs, which is often more severe as compared to DM and PM. The involvement of other organ systems, such as the skin, is very rarely seen [10]. The medical research council (MRC) scale is the most widely used to grade the muscle strength in these patients, which usually shows maximal involvement of the hip flexor muscles. Patients with IMNM have the highest elevations of CK among the various forms of muscle enzymes, with the median peak around 4700 U/L, in contrast to the median peak in DM, which is around 700 U/L [2]. Based on the autoantibodies present, IMNM is classified into three distinct subtypes - anti-HMGCR myopathy, anti-SRP myopathy, and antibody-negative IMNM [11]. Muscle MRI has only limited value in diagnosing necrotizing myopathy but demonstrates patchy involvement with muscle edema, muscle atrophy, and fatty replacement of muscle with minimal fascial edema [12]. The overall extent of muscle involvement is more in IMNM as compared to DM/PM/IBM. Electromyography may be adjunctive to diagnosis. The histopathological hallmark of IMNM is the presence of randomly distributed necrotic muscle fibers and fibers in various stages of regeneration but in the absence of or sparse mononuclear infiltrates. Macrophages are the predominant mononuclear cell type, whereas T and B cells are virtually absent. There is usually multifocal upregulation of class I MHC and deposition of MAC on the sarcolemma of non-necrotic muscle fibers [13].

Cancer-associated myositis is defined as malignancy occurring within three years before or after the diagnosis of myositis. Paraneoplastic necrotizing myopathy has been very rarely described, and the earliest report of four such cases was by Levin et al. in 1998 [4]. A review of the literature of all cases until 2011 by Gultekin reported only about 50 cases in total [14]. A longitudinal observational study on 115 patients with necrotizing myopathy by Allenbach et al., published in 2016, reported cancer in the three different subgroups of IMNM - 28.6% antibody negative, 17.3% anti-HMGCR, and 8.1% anti-SRP. Five cases of breast cancer were reported in their study, all diagnosed after the diagnosis of myopathy. There was no predominance of any specific cancer type [15]. IMNM explicitly associated with breast cancer has been described in detail only in a case report by Silvestre et al., published in 2009. Their patient presented with symptoms approximately 10 months after the diagnosis of Stage IIIC invasive ductal carcinoma, which was treated with chemotherapy and radiation. The chemotherapy regimen was later modified due to the persistent tumor burden [16]. Cutaneous manifestations in IMNM have been rarely described. A retrospective study of 15 patients with NM by Mahnaz et al. reported a rash in one patient with high-grade serous carcinoma of the ovary [17]. Authier et al. describe a case of painful myopathy related to necrotizing myopathy associated with the cutaneous signs of dermatomyositis [18]. Some of these characteristics matched those of our patients'.

The pathophysiology of paraneoplastic IIMs has not been clearly elucidated. There are, however, theories that have been proposed to explain this phenomenon. The classical hypothesis of paraneoplastic neurological diseases is the de novo expression of non-exposed nervous tissue antigen by the tumor tissue, which leads to the development of an immune response that also concomitantly attacks the nervous system [14]. Cancers may present self-antigens, including myositis auto-antigens, at a higher than normal frequency because of the monoclonal and rapidly expanding nature of tumor cells [19]. T-cell activation and survival likely contribute to the pathogenesis of paraneoplastic syndromes [7]. There has been recent mention of a new antibody against transcriptional intermediary factor 1-gamma (anti-155/140 antibody) that has been associated with a significant percentage of adult myositis patients [20]. The presence of this antibody in IMNM has not been adequately studied and might provide an insight into its pathophysiology in the future.

## Conclusions

Recent studies have shown the association of IMNM with malignancy. This emphasizes the need to screen all patients with features of necrotizing myopathy for underlying malignancy. Although systemic manifestations in IMNM are rare, the presence of dermatological features, such as a facial rash, should not eliminate IMNM from the differential and warrants a broader investigation. There is an increased emphasis on antibody testing required to make a diagnosis of IMNM as well as to classify it into subtypes. Therefore, testing for necrotizing myopathy is recommended in all cases, whenever feasible. 
